# Possible Link between SARS-CoV-2 Infection and Parkinson’s Disease: The Role of Toll-Like Receptor 4

**DOI:** 10.3390/ijms22137135

**Published:** 2021-07-01

**Authors:** Carmela Conte

**Affiliations:** Department of Pharmaceutical Sciences, University of Perugia, via Fabretti, 06123 Perugia, Italy; carmela.conte@unipg.it

**Keywords:** Parkinson’s disease, SARS-CoV-2, COVID-19, toll-like receptor 4, synuclein, neuroinflammation

## Abstract

Parkinson’s disease (PD) is the most common neurodegenerative motor disorder characterized by selective degeneration of dopaminergic neurons in the *substantia nigra pars compacta* (*SNpc*) of the midbrain, depletion of dopamine (DA), and impaired nigrostriatal pathway. The pathological hallmark of PD includes the aggregation and accumulation α-synuclein (α-SYN). Although the precise mechanisms underlying the pathogenesis of PD are still unknown, the activation of toll-like receptors (TLRs), mainly TLR4 and subsequent neuroinflammatory immune response, seem to play a significant role. Mounting evidence suggests that viral infection can concur with the precipitation of PD or parkinsonism. The recently identified coronavirus named severe acute respiratory syndrome coronavirus 2 (SARS-CoV-2) is the causative agent of ongoing pandemic coronavirus disease 2019 (COVID-19), responsible for 160 million cases that led to the death of more than three million individuals worldwide. Studies have reported that many patients with COVID-19 display several neurological manifestations, including acute cerebrovascular diseases, conscious disturbance, and typical motor and non-motor symptoms accompanying PD. In this review, the neurotropic potential of SARS-CoV-2 and its possible involvement in the pathogenesis of PD are discussed. Specifically, the involvement of the TLR4 signaling pathway in mediating the virus entry, as well as the massive immune and inflammatory response in COVID-19 patients is explored. The binding of SARS-CoV-2 spike (S) protein to TLR4 and the possible interaction between SARS-CoV-2 and α-SYN as contributing factors to neuronal death are also considered.

## 1. Introduction

According to the World Health Organization (WHO), Parkinson’s disease (PD) is the second most common neurodegenerative disease in the world, after Alzheimer’s disease (AD), and one of the most common causes of neurological disability with a high social impact [1]. The prevalence of PD is about 10 million people worldwide, and it is estimated that there will be about 13 million people with PD by 2040 [2]. PD is a chronic, progressive, age-related neurodegenerative disease clinically characterized by motor symptoms, such as bradykinesia, rigidity, tremor at rest, slow movements, and postural instability [3]. Motor symptoms are the consequence of the progressive loss of neuromelanin-containing neurons, especially dopaminergic neurons (DA) in the *substantia nigra pars compacta* (*SNpc*) and dopamine depletion in the striatum, as confirmed by post-mortem study of clinically diagnosed PD patients [4,5]. The pathological hallmark of PD is represented by the accumulation of cytoplasmic inclusions such as Lewy bodies (LB) and Lewy neurites rich in α-synuclein (α-SYN) [6,7,8].

Albeit a number of genetic and environmental risk factors have been characterized, the cause(s) of PD are still unknown. In the last decades, studies have suggested the association between certain viral infections and acute and chronic parkinsonism. Most virus species include herpesvirus, Coxsackie, Japanese encephalitis B, Epstein Barr, Human Immunodeficiency Virus (HIV), and western equine encephalitis [9,10,11,12,13,14,15,16,17].

In the CNS, specialized innate immune sentinels, such as microglia, macrophages, dendritic cells, and astrocytes detect and clear viral agents by activating a robust immune response. However, many viruses use different strategies to elude immune system, cross the blood–brain barrier (BBB), and directly enter the nervous system. For example, viruses can enter the CNS via a “Trojan horse” mechanism using infected leucocytes as a vehicle for the passage from the blood through the BBB. [18]. Viruses may cause the disruption of the BBB integrity to gain access to the CNS [19]. Viral invasion of neural tissues can initiate inflammatory signaling by local primed immune cell recruitment, resulting in the release of abundant levels of an array of proinflammatory cytokines/chemokines that in turn can disrupt the BBB and increase its permeability [20].

The connection between systemic inflammation, PD, and neuroinflammation has been largely elucidated and a number of studies suggest that Toll-like receptors (TLRs), mainly TLR2 and 4, participate in the pathogenesis of PD as promoters of immune/neuroinflammatory responses that precede both motor and non-motor symptoms. The overexpression of TLR4 has been found in circulating monocytes of PD patients, in B cells, and in the caudate/putamen [21,22,23,24]. Studies in animal models of PD reported the potential role of TLR4 in mediating biochemical changes as well as dopaminergic cell death and α-synuclein accumulation in the midbrain [25,26]. Moreover, TLR4 has been found to play a critical role as a mediator of the neurotoxicity induced by α-synuclein oligomers [27].

Coronavirus disease (COVID-19) is an ongoing pandemic caused by a novel RNA (32 Kb genome) virus, namely, severe acute respiratory syndrome coronavirus 2 (SARS-CoV-2), whose worldwide cases passed 160 million. COVID-19 deaths passed three million worldwide, and unfortunately these numbers are destined to further increase [28]. It is a highly transmissible virus strain of the recently discovered coronavirus in China and is transmitted primarily via respiratory droplets. It shares genetic identity with SARS-CoV and MERS-CoV [29,30]. It is composed of four major proteins named the spike (S), envelope (E), membrane (M), and nucleocapsid (N) [31]. All these proteins are notably implicated in viral infection, proliferation or host cell pathogenesis and are therefore predictable as the potential targets for vaccine or drug development.

SARS-CoV-2 acts through the binding between the viral receptor S protein and different glycoprotein receptors on the cell surface. The virus S protein is a flexible and instable glycosylated complex. Recently, high-quality and stabilized forms of the trimeric S protein have been produced for use in vaccines and diagnostic tests [32].

A priming step of virus infection is represented by the cleavage of S protein by the transmembrane serine protease 2 (TMPRSS2) [33]. Then, cellular endocytosis occurs and RNA replication stages can take place. The main protein target on the cell surface is the angiotensin converting enzyme 2 (ACE2), an enzyme involved in converting angiotensin II to angiotensin. ACE2 is highly expressed in a wide variety of human tissues, including the brain [34,35,36,37,38].

SARS-CoV-2 has potential affinity to other candidate functional receptors such as dipeptidyl peptidase 4 [39], sialic acid residues on surface of airway cells and neurons [40], and lectin CD209L [41].

The respiratory tract is the first site that can be infected by SARS-CoV-2. If the virus is not cleared by the immune response, it reaches the lower respiratory tract and rapidly can be transmitted through droplets in a human-to-human manner [42]. COVID-19 includes a wide spectrum of clinical manifestations ranging from respiratory illness, with symptoms such as bronchitis, pneumonia, asthma, chronic obstructive pulmonary disease, and severe respiratory distress syndromes to multiorgan severe inflammation [43,44]. The high levels of cytokines secreted during the multisystem inflammatory syndrome can cause septic shock, sometimes fatal for the patients. Importantly, host response is fundamental to challenge the uncontrolled inflammatory response and plays a crucial role in susceptibility to SARS-CoV-2-mediated diseases [45].

Epidemiologic studies demonstrate that, unlike polio and others virus that evocate more severe complications for the young, SARS-CoV-2 infection is a zoonosis that affects older people, suggesting that age-associated chronic inflammation may occur with the development of disease [46].

A higher pathogenicity and death rate have been observed in particularly vulnerable and frail subjects and/or those affected by chronic inflammation or multiple comorbidities including diabetes, obesity, cardiovascular diseases, and immunosuppression [47,48,49]. Recent genome-wide association studies identified potential genetic factors involved in the development of COVID-19 [50]. Although the precise factors determining SARS-CoV-2 infection are poorly understood, systemic immune and hyperinflammatory responses play a major role. For example, it has demonstrated that the interaction between ACE2 and the S protein stimulates NF-kB activation, which results in the release of proinflammatory cytokines in infected cells [51]. The cytokine storm affecting hospitalized patients with severe COVID-19 is responsible for multiple organ failure and includes the nervous system.

This review focuses on the suspected intriguing link between SARS-CoV-2 infection and the pathogenesis of PD with particular attention on the potential involvement of TLR4 as a common pathway during harmful inflammatory responses in both diseases.

Although TLR4 signaling represents one of a series of immune and inflammatory factors found activated during both PD and COVID 19, the strong interaction of TLR4 with the SARS-CoV-2 S protein as well as the possible binding between SARS-CoV-2 and α-SYN suggest triggering mechanisms of neurodegenerative processes underlying PD, which deserve to be elucidated.

## 2. Neuroinflammation 

Neuroinflammation is a complex process able to activate the innate immune response in the central nervous system (CNS), associated not only with neurodegenerative diseases but also with infections and trauma. For a long time, the CNS has been considered an immunologically privileged site because the lack of cells capable of presenting the antigen in the brain parenchyma, the absence of lymphatic drainage, and the presence of BBB. Over the last 30 years, this concept has been challenged, and many reports show active cooperation between the CNS and immune system despite the physical barrier [52,53].

A growing body of evidence indicates that neuroinflammation is involved in the pathogenesis of several neurodegenerative diseases including PD [54,55,56,57], Alzheimer’s disease (AD) [58], amyotrophic lateral sclerosis [59], Huntington’s disease, and virus-associated dementia [60,61]. Neuroinflammation implicates the recruitment of immune and non-immune cells at the site of injury or infection, such as resident macrophages, i.e., microglia, aside from astrocytes, endothelial cells, infiltrating T-lymphocytes, α-SYN reactive T-lymphocytes, and major histocompatibility complex (MHC) class II-positive microglia [62,63,64,65]. Microglia are the most abundant populations in the brain parenchyma and represent the major cell type involved in neuroinflammation However, sophisticated neuron–microglia–astrocyte crosstalk also plays a critical role in the immune response [66,67,68].

Although no standardized morphological classification exists, the mature brain microglial cells are generally quiescent or “resting” and exhibit a ramified shape with fine extending protrusions. They also are equipped with a wide range of receptors, comprising colony-stimulating factor 1 receptors that continuously scan the surrounding environment and control important functions such as synaptic activity, clearance of cell debris, and cerebral homeostasis [69,70,71,72].

Immunological and inflammatory stimuli or invading agents, including viruses, can promote phenotype switching between a highly ramified morphology and a large ameboid-like shape with short branches typical of activated microglia [73,74,75]. This switching triggers a complex cascade of events whose magnitude depends on the intensity and duration of the stimulation [76].

While acute insults evoke protection and healing of the brain tissue, chronic stimulation causes a sustained inflammatory response resulting from the acquisition of an array of functions by microglia associated with upregulation of a large number of receptor types, such as cytokine receptors, TLRs or cell adhesion molecules.

The activation of TLRs initiates intracellular signaling pathways that culminate with the release of interleukins, interferons, chemokines, and other toxic compounds. Chemokines attract more microglia that further contribute to propagate the neuroinflammatory process and activate the apoptotic machinery [77].

Whether neuroinflammation is a consequence or a cause of nigral cell loss in PD is still unclear. Certainly, several neurodegenerative diseases, including PD, are known to impact the switch of microglia from the neuroprotective to the neurotoxic phenotype [78,79,80,81]. Sustained gliosis seems to be the prominent pathological feature of PD that is associated with loss of dopaminergic neurons in the *SNpc* [82,83,84]. Importantly, the degeneration of the dopaminergic neurons could be caused not only by glial response but also by the loss of a supporting role by resting microglia following switching to an activated status. Neuroinflammation in PD is also accompanied by microglia uptake and phagocytosis of neuromelanin released by degenerated DA neurons into the extracellular compartment that contribute to the acceleration of the neurodegenerative process.

In 1988, McGeer et al. [85] showed the presence of reactive microglia in the *SNpc* of human post-mortem brain tissues. High levels of interleukin-1β (IL1-β), IL-6, TNFα, interferon γ (IFNγ), cyclooxygenase type 2 (Cox-2), nitric oxide synthase (NOS), infiltrating peripheral immune cells, and ROS were found in post-mortem PD brains as well as in cerebrospinal fluids of patients with PD. A recent postmortem study on demented PD cases revealed the upregulation of neuroinflammatory markers and stated that α-SYN pathology and microglia activation play a critical role [86,87].

## 3. Toll-Like Receptors

TLRs are type I transmembrane glycoproteins with an extracellular leucine-rich repeat motif and a cytoplasmic Toll/IL-1 receptor (TIR) signaling domain, similar to the interleukin-1 receptor domain (IL-1R) [88]. Both IL-1R and TLRs initiate downstream signaling, leading primarily to the activation of the transcription factor NF-kB, a key regulator of inflammatory response [89]. TLRs belong to the complex pattern recognition receptors (PRRs) expressed in immune and non-immune cells, including neurons and glia, which are involved in regulating the innate immune system and inflammatory response by producing inflammatory cytokines and other mediators [90]. These processes prime immediate host-defense responses crucial for the clearance of infecting agents and for the following adaptative immune responses [91].

**Figure 1:**
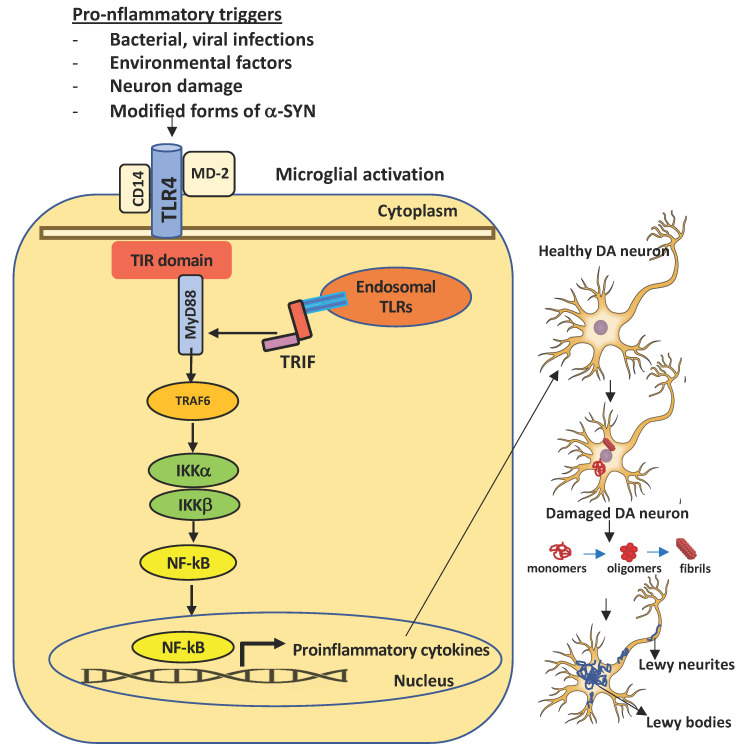
Schematical representation of TLR4 activation in microglia and consequent dopaminergic neuronal damage in the *substantia nigra*. A number of pro-inflammatory stimuli can promote the activation of the intracellular TLR4 signaling pathway in microglia. Upon infection or injury, DAMPs or PAMPs released in the extracellular milieu via exocytosis from neighboring neurons are sensed by microglial TLR4 for degradation and clearance. If compromised or if prolonged, this process can lead to further inflammatory signaling in which the engagement of myeloid differentiating primary response gene 88 (MyD88) or TIR-domain containing adapter inducing interferon β (TRIF) provokes the activation of downstream signaling cascades that lead to the proteasomal degradation of kB inhibitors (IkB), release, and nuclear translocation of the NF-kB (nuclear factor kappa-light-chain-enhancer of activated B cells) transcription factor. The transcriptional activation of specific genes induces the release of proinflammatory cytokines, that in turn can cause neuronal damage, pathological modification of α-SYN (monomers, oligomers, and fibrils, truncated and phosphorylated forms), and aggregation in Lewy and neurites bodies—the pathological hallmarks of degenerating neurons in Parkinson’s disease. Endosomal TLRs sense bacterial or viral nucleic acids.

TLRs are highly specialized in sensing invading bacteria, viruses, parasites, and cell debris [92,93]. The TLR family comprises 11 members (TLR1–TLR11) in human and 12 (TLR1–TLR9, TLR11–TLR13) in mouse. They are localized on the cell surface as well as in intracellular compartments such as the endoplasmic reticulum, endosome, lysosome, or endolysosome, which normally respond to viral nucleic acids. However, they survey for the presence of structural motifs in a wide array of invading microorganisms, named pathogen-associated molecular patterns (PAMPs), in the extracellular space and within endocytic compartments. TLRs also sense endogenous damage or danger molecular patterns (DAMPs), also known as alarmins, released by damaged cells and injured tissues or derived from apoptotic and necrotic cells [93]. The activation of neuro-inflammatory machinery starts with the binding between DAMPs/PAMPs and TLRs and advances with the dimerization of TLRs and the subsequent interaction with adaptor proteins such as Myeloid Differentiation Primary Response Gene 88 (MyD88) and Toll/interleukin-1 receptor-like (TIR)-domain containing adapter-inducing interferon-β (TRIF) domain. This cascade of events leads to the recruitment of others complexes, such as IL-1R-associated kinase (IRAK) and MAP kinases, and the nuclear translocation/activation of transcription factors, including NF-kB, that ultimately trigger the downstream overexpression of pro-inflammatory genes and cell degeneration [94] (Figure 1). Among the 11 human TLRs members, TLR4 seems to play a critical role in the development and progression of neurodegenerative diseases, including PD [95]. A broad variety of molecules are recognized by TLR4. They include (1) exogenous natural ligands such as canonical lipopolysaccharide (LPS) from gram-negative bacteria, viruses, fungi, and mycoplasmas); (2) extracellular matrix ligands (hyaluronan, biglycan, fibronectin, and heparan sulphate); (3) intracellular and secreted endogenous ligands (heat shock proteins, defensins, S100 proteins, and amyloid β) [96].

## 4. TLR4

Analysis of transcriptomic data from human postmortem control brains reveals ubiquitous expression of TLR4 throughout the brain, with their expression being higher in the *substantia nigra* and the putamen. Increased TLR4 protein levels were found in peripheral immune cells as well as in the *substantia nigra* and caudate/putamen of PD cases [24,97].

Additional studies in experimental models of PD and α-synucleinopathies demonstrated the important role of TLR4 in α-synucleinopathies and the constitutive expression in microglia and the up-regulation in the *substantia nigra* [68,69,97,98,99,100].

TLR4 is expressed by two types of non-neuronal supportive cells: the CNS residential macrophages or microglia and the macroglial cells such as astrocytes.

Genetic studies show that polymorphism in the TLR4 genes is linked with the risk of PD [101,102].

In the brain, TLR4-mediated signaling pathways have been implicated in the pathogenesis of PD [103]. Its stimulation causes the production of elevated levels of neurotoxic inflammatory cytokines that damage nigral dopaminergic neuron [104]. α-SYN as well as neuromelanin and other molecules released by damaged neurons can act as ligands for TLRs, including TLR4, and generate a sustained immune response that initiates inflammatory processes implicated in PD [105,106].

Drouin-Ouellet and co-workers [23] showed that the overexpression of α-SYN induced a critical modulation of TLR4 signaling in the blood and brain of both experimental models and PD patients. This triggers an immune/inflammatory response that culminates with neuronal death and negatively impacts motor symptoms. However, a study supported a role for TLR4 in mediating the clearance of α-SYN, suggesting an innate neuroprotective mechanism [107]. Therefore, both neuroprotective and detrimental roles of TLR4 in PD have been suggested: acute stimuli such as posttranslational modification of α-SYN can be a trigger for TLR4 microglial activation and protein clearance. Conversely, chronic inflammation, from inside or outside the nervous system, may promote the imbalanced activation of TLR4 signaling and escalation of inflammatory response, which may contribute to the pathogenesis of PD [108,109]. A large number of molecules, including several forms of α-SYN (monomer, oligomers, and fibrils, truncated and phosphorylated), released from neighbouring neurons in the extracellular milieu, act as ligands for TLR4 and undergo phagocytosis, degradation, and clearance. If comprised, this mechanism can lead to further inflammatory signals that occur with the formation of aggregated forms of α-SYN in the Lewy bodies of *substantia nigra*.

The formation of the complex TLR4-myelod differentiation 2 (MD2) on the cell surface, as well as the presence of LPS-binding proteins and cluster of differentiation 14 (CD14), is required for the recognition of ligands and the activation of intracellular pathways. The dimerization of TLR4-MD2 induces conformational changes that endorse the recruitment of adaptor proteins containing Toll/interleukin-1 receptor-like (TIR) domains. This is essential to start two possible intracellular signal pathways: the MyD88-dependent pathway culminating with the release of proinflammatory cytokines and the MyD88-independent pathway leading to the production of the type I interferons [110].

The engagement of MyD88 and MyD88-adaptor-like (MAL) protein induces a cascade of downstream events including the phosphorylation of IL-1R-associated kinases (IRAKs), the association of TNF-receptor-associated factor 6 (TRAF6), and the activation of transforming growth factor β-activated kinase 1 (TAK1). Many other adaptor proteins are involved and include TAK1-binding protein 2 (TAB2) and TAK1-binding protein 3 (TAB3). TAK1, in turn, activates two different kinase types: the mitogen-activated protein kinases (MAPKs), which comprise JUN N-terminal kinase (JNK), p38, extracellular signal-regulated kinases (ERK1/2), and the IkB kinase complex (IKK). The final response is the activation of nuclear factor kappa-light-chain-enhancer of activated B cells (NF-kB) and activator protein-1 and the abundant secretion of proinflammatory cytokines [111]. Instead, the activation of the MyD88-independent pathway is started by internalization of the TLR4-MD2 complex by CD14 and the recruitment/activation of adaptor proteins TRIF, TRIF-related adaptor molecule (TRAM), and TRAF3 followed by IFN regulatory factor 3 (IRF3) nuclear translocations. These events culminate with the production of type I IFNs [112]. Sustained activation of these signals attracts macrophages, natural killer, and other cells and may result in the toxic accumulation of ROS and RNS in different cell types, including neuronal cells [113,114] (Figure 1).

## 5. SARS-CoV-2 Inflammatory Response: Role of TLRs

Currently, the identification of the precise mechanism underlying the cytokine storm in hospitalized patients with severe COVID-19 is essential. The severity of COVID-19 is associated with the excessive inflammatory innate response and dysregulated adaptive host immune defense. In this context, TLRs can have a dual role. In fact, the activation of the innate immune system through TLRs can be the first line of defense against invading viruses and can support the elimination of viruses. However, prolonged and dysregulated activation may contribute to the onset of the hyperinflammation and poor outcome of clinical manifestations of COVID-19 [115,116].

Research on influenza viruses suggests that the expression of cytokines by lung epithelial, macrophages, and dendritic cells is the result of the activation, by viral PAMPs, of intracellular or extracellular TLRs. The main viral PAMPs are proteins, double strand RNA (dsRNA), single strand RNA (ssRNA), and CpG DNA, which are recognized by distinct TLRs, including TLR2, TLR3, TLR7, TLR8, and TLR9 [117]. Upon ligand recognition, all TLRs except TLR3 recruit MyD88. TLR3 and TLR4 also recruit the adapter protein TRIF. The MyD88-dependent and TRIF cascades initiate the nuclear translocation of NF-κB, IRF3, and IRF7 transcription factors that culminate with the upregulation of inflammatory cytokines/chemokines (through NF-kB) and IFN genes (through both IRF3 and IRF7) [118].

Systemic inflammation in patients with COVID-19 has been associated with elevated serum levels of pro-inflammatory cytokines and chemokines such as IL-2, IL-7, IL-8, IL-10, IP-10 (interferon-γ-inducible protein), MCP-1 (monocyte chemoattractant protein), MIP-1α (macrophage inflammatory protein 1 alpha), and TNFα. Additionally, high levels of IgG and a high neutrophil-to-lymphocyte ratio have been observed [116,119].

Recently, TLRs signaling pathways have been recognized as accessory factors that may occur with the COVID-19 pathogenesis. The main members include TLR2, TLR3, TLR4, TLR6, TLR7, TLR8, and TLR9, with beneficial and harmful effects towards SARS-CoV-2 infection. The activation of TLR7/8 has been found to evoke a strong pro-inflammatory response during acute lung injury [120]. Furthermore, the binding of SARS-CoV-2 PAMPs to the extracellular domain of human TLR1, TLR4, and TLR6 seems to be crucial for COVID-19 immunopathogenesis [121,122].

The interaction between the SARS-CoV-2 S protein and cell surface TLRs has been investigated by molecular docking studies. The results have demonstrated a significant binding of the S protein to TLR1, TLR4, and TLR6, especially TLR-4, suggesting a potential mechanism of the cytokine cascade [121].

## 6. Interaction between the SARS-CoV-2 S protein and TLR4

Although the host genetic background powerfully influences the requirement for TLR4-mediated signals during SARS-CoV-2 infection, TLR4 activation seems to represent one of the critical steps for host–virus general interaction [122]. It has been demonstrated that TLR4 deficiency can induce resistance to acute manifestations that accompany the viral attack, suggesting an important role of TLR4 signaling in the profuse pro-inflammatory cytokine storm [123].

Structural analysis by molecular docking revealed that the strongest binding was observed between the native spike glycoprotein and human TLR4 compared with other TLRs present in the human cells [121]. The spike protein is a surface-exposed homo-trimeric transmembrane, heavily glycosylated complex with high binding affinity for the ACE2 receptor of human host cells 32. Another recent study found an interaction between the S protein and *Escherichia coli* LPS, the well-known activator of TLR4 [124].

A robust binding was also observed with the TLR4 accessory proteins CD14 and MD-2 that are overexpressed in inflammatory circumstances [125]. Interestingly, multi-epitopic regions of the SARS-CoV-2 S protein have been found to interact with the TLR4/MD2 complex. Therefore, it could act as a potent peptide vaccine candidate against COVID-19 [125]. Studies using surface plasmon resonance confirm the direct binding between the SARS-CoV-2 trimer and TLR4 in THP-1 cell lines, neutrophils, primary bone marrow-derived macrophages, and peritoneal macrophages [126]. Ziegler et al. [127] showed that the interaction between the S protein and TLR4 induced the overexpression of interferon-stimulated genes, which in turn provoked the upregulation of ACE2. By this ingenious strategy, the viral entry is facilitated.

**Figure 2:**
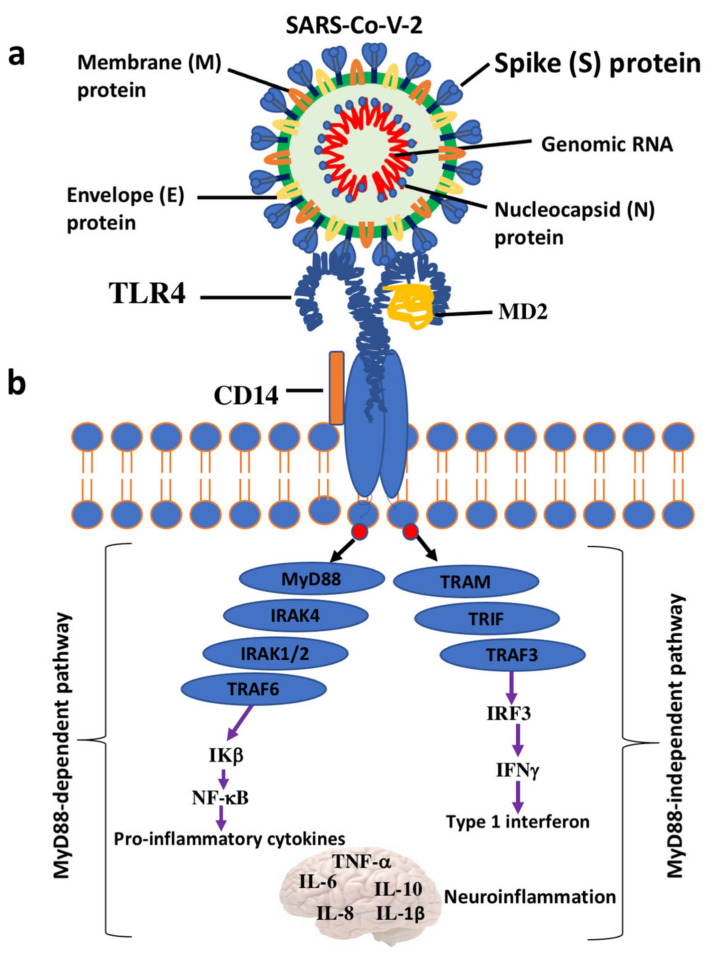
Toll-like receptor 4 (TLR4) signaling cascade resulting from the interaction between TLR4 and SARS-CoV-2 S protein. (**a**). Schematic diagram of the primary structural proteins of SARS-CoV-2.(**b**). The interaction between TLR4 and the SARS-CoV-2 S protein can trigger an intracellular TLR4 signaling cascade that can be one of the factors leading to the cytokine storm and neuroinflammation in severe COVID-19 patients. SARS-CoV-2: Severe Acute Respiratory Syndrome Coronavirus 2; Coronavirus disease. CD14: cluster of differentiation 14; MD2: myeloid differential protein-2; MyD88: myeloid differentiating primary response gene 88; NF-kB: nuclear factor kappa-light-chain-enhancer of activated B cells; IRAK: interleukin-1 receptor-associated kinases; TRIF: TIR-domain containing adapter inducing interferon β; TRAM: TRIF-related adaptor molecule; TRAF: tumor necrosis factor receptor-associated factor; IFN: interferon.

Although requiring confirmation, hydrogen bonds and hydrophobic interactions seem to assist the formation of the TLR4-spike complex. Together with ACE2, this event may be crucial for the activation of TLR4 signaling, NF-kB translocation, and hyperinflammation accompanied by the release of the TNF-α, IL-6 cytokines and more robustly IL1β [128]. Further studies are necessary to identify the specific TLR4 motifs involved. No interaction was observed by using the N-terminal or the receptor-binding domains of the spike protein [129] (Figure 2).

## 7. TLR4 and PD Pathogenesis

Both COVID-19 and PD are associated with dysfunction of the immune system and neuroinflammation, although the precise mechanisms remain unknown. The overexpression of TLR4 in PD and in COVID-19 patients together with high levels of many other inflammatory mediators could explain the link between these diseases. TLR4 downstream signaling molecules and inflammasome-related proteins were found to be upregulated in the blood cells from COVID-19 and PD patients [130,131]. The binding of the SARS-CoV-2 S protein to TLR4 on the neuron surface could represent a trigger for neurodegeneration, especially for the dopaminergic neurons in the nigrostriatal system, which express TLR4. As result, the recruitment of intracellular signaling pathways, such as MYD-88 together with upregulation of inflammatory signaling molecules could lead to the NF-kB-induced release of cytokines, reactive oxygen species (ROS), and reactive nitrogen species (RNS), and finally culminate with neurodegeneration [132].

Dysregulation of TLR4 signaling has been shown to play a role in the initiation and/or progression of PD. Notably, a number of DAMPs are released as a result of neuronal injury in PD, such as misfolded and aggregated α-SYN. The latter alters TLR4 expression and can act as a TLR4 agonist, further exacerbating the inflammatory status through activation of surrounding neurons and microglia cells [133].

The increase in systemic TLR4 agonists such as oxidized lipoproteins and phospholipids, heat shock proteins, extracellular matrix protein, and many other molecules secreted during COVID-19 can act as PAMPs for TLR4 and contribute to further damage the host brain parenchyma. Overall, the permanence of the virus for a long time in the CNS could be a factor predisposing patients to neurodegenerative processes as in PD.

## 8. Neuroinvasive Potential of SARS-CoV-2 and PD Pathogenesis

The concept of “Neuro-COVID-19” is being mentioned increasingly, but whether neurological manifestations in patients with COVID-19 are due to direct invasion of the virus or result from SARS-CoV-2-dependent neuroinflammatory response remains speculative.

Historically, viral infections were associated with parkinsonism. Viral agents can act as an initiator of parkinsonism as well as of other neurological complications [134]. For example, viruses such as influenza, Coxsackie, Japanese encephalitis B, western equine encephalitis, herpes, and viruses related to acquired immunodeficiency disorder, including HIV, are associated with both acute and chronic parkinsonism [10,135]

The trans-synaptic transfer of CoV and avian bronchitis viruses, as well as of other respiratory viruses, has been widely documented [136,137]. Evidence showed the ability of the SARS virus to penetrate into the CNS and cause neuropathy and gliopathy [138]. SARS genome sequences were detected in the hypothalamus and cortex of SARS autopsies with clear signs of edema and neurodegeneration [139].

The respiratory distress caused by SARS-CoV-2 infection that emerged in December 2019 occurs in about 73% of COVID-19 hospitalized patients by a number of neurological signs associated with dysfunction of the central nervous system [140], peripheral, and enteric [141] nervous systems. The symptoms include olfactory, gustatory, dizziness, headache, dizziness, confusion, encephalitis, stroke, anorexia, myalgias, and gastrointestinal disorders such as nausea and vomiting. Severe COVID 19 patients under intensive treatment exhibit cerebrovascular disease, consciousness state, and skeletal muscle injury [142,143].

SARS-CoV-2 infects cells through the interaction between its S protein previously cleaved by TMPRSS2 and ACE2. The neuroinvasive propensity of SARS-CoV-2 is probably facilitated by high expression levels of ACE2 and TMPRSS2 in neurons, astrocytes, and oligodendrocytes that, together with TLR4 activation, can predispose patients to α-SYN aggregation, neurodegeneration, and PD pathogenesis [144]. As shown in the Allen Human Brain Atlas, ACE2 mRNA is widely distributed throughout the brain with notable strong expression in areas such as the cortex, striatum, hypothalamus, medulla, and *substantia nigra* [145]. ACE2 receptors are expressed in neurons, astrocytes, and oligodendrocytes in the *substantia nigra* and olfactory bulb [146]. However, ACE2 may not be the only binding site involved in brain uptake [147].

SARS-CoV-2 can circumvent the host immune response and spread within the CNS directly through the olfactory nerves [148] or similar to other viruses, can reach the brainstem through the vagus nerve, infect neurons of different areas, and induce a marked systemic pro-inflammatory response associated with the overproduction of cytokines [149,150,151,152,153]. Using a human brain organoid model, Song et al. [148] showed that SARS-CoV-2 can infect cells of neural origin and cause death of nearby cells. SARS-CoV-2 particles were also detected in the cortical neurons from postmortem autopsies of patients who died from COVID-19 as well as in the cerebrospinal fluid of SAR-CoV-2-positive cases [154].

**Figure 3:**
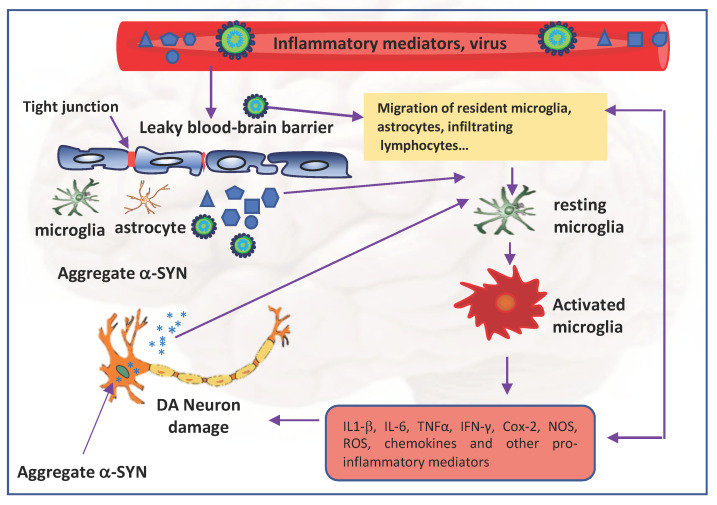
SARS-CoV-2 neuroinvasion and neuropathogenesis. SARS-CoV-2 may cause the disruption of the BBB integrity to gain access to the CNS. Viral invasion initiates inflammatory signaling involving the activation of microglia and release of abundant levels of proinflammatory cytokines/chemokines, which in turn can disrupt the BBB and increase its permeability. The inflammatory response can cause the neuronal accumulation of α-SYN and neuronal damage. The aggregates of α-SYN released from the neurons can induce microglia activation and initiate a vicious circle. IL: interleukin; TNFα: tumour necrosis factor alpha, IFN-γ: Interferon-gamma; Cox-2; cyclooxygenase-2; NOS: nitric oxide synthase; ROS: reactive oxygen species.

The impact that COVID-19 might have on PD pathogenesis is debated at present. PD is associated with changes in the CNS immune response, microglia and oligodendroglial activation, upregulation of histocompatibility class II, and pro-inflammatory cytokine overproduction. SARS-CoV-2 infection may be a predisposing factor for an abundant immune response. The cytokine storm during COVID-19 can cause the breakdown of the BBB and lead to virus entry and immune cell infiltration [155]. This directly can cause neuronal death and escalation of patient care towards severe neurological complications characterizing all synucleinopathies, including PD [156,157,158,159] (Figure 3).

Parkinsonism has been reported following COVID-19. Although Parkinson’s disease was not diagnosed, functional nigrostriatal neuroimaging was abnormal in some COVID-19 cases, therefore presuming dopaminergic nigrostriatal impairment [160,161].

Moreover, some of the most common non-motor symptoms of PD are evident in COVID-19 patients and comprise anosmia/iposmia, gastrointestinal symptoms, ageusia, fatigue, and painful limbs [162,163]. These symptoms antedate the pathological deposition of α-SYN and the appearance of motor symptoms.

The possible trans-synaptic spread via the olfactory, lingual, and glossopharyngeal nerves of SARS-CoV-2 could explain the featured symptoms such as hyposmia and ageusia in many COVID-19 patients [164,165].

The presumed degeneration of dopaminergic nigrostriatal nerves could derive from immune activation in the olfactory system (without direct virus entry) that, associated with other toxic stress and the inability to activate neuroprotective responses, might promote α-synuclein misfolding, aggregation, and neurodegeneration.

Similar to PD, increased levels of IL-6 have been also found in COVID-19 patients, indicating an impact of inflammation on the progression of non-motor impairment [166,167,168].

**Figure 4:**
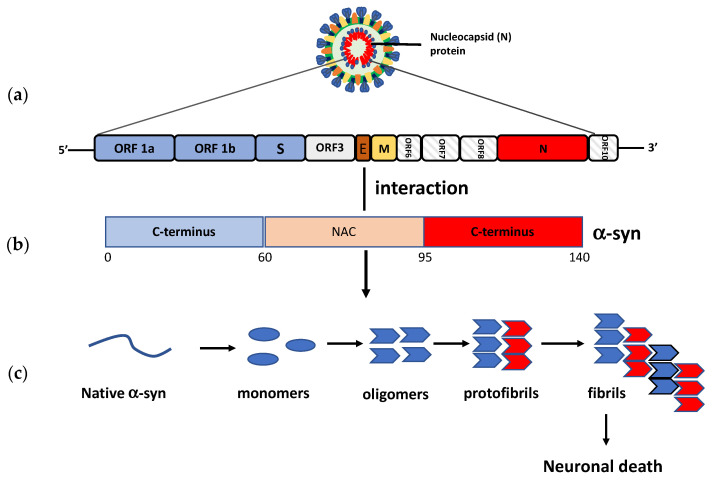
Possible direct interaction between the spike protein and α-sinuclein. (**a**) SARS-CoV-2 genome organization. Open Reading Frames (ORFs); Envelope (E); Nucleocapside (N); Membrane protein (M). (**b**) Schematic depiction of α-synuclein structure. N-terminus, NAC (non-amyloid-β component) region, and C-terminus are coloured blue, pink and red, respectively. (NAC). (**c**) The possible direct interaction between SARS-CoV-2 and α-SYN could start a conformational shift of the monomeric protein and accelerate the formation of toxic multimeric protein species, such as oligomers/protofibrils and fibril aggregates, resulting in neuronal death.

α-SYN is the most important protein implicated in PD. Similar to West Nile virus and SAR-CoV-1, SARS-CoV-2 infection could lead to α-SYN upregulation in attempts to prevent viral replication and neuroinvasion [169,170]. However, abundant and protracted systemic inflammation could disturb the host cell proteostasis and protein quality control system, contributing to the pathological modification of α-SYN that can evolve with the formation of fibrillar structures. Although confirmation is needed, recent findings suggest that the interaction between the SARS-CoV-2 N protein and α-SYN could accelerate the protein aggregation into amyloid fibrils, leading to propagation and widespread neurodegeneration [171] (Figure 4).

**Figure 5:**
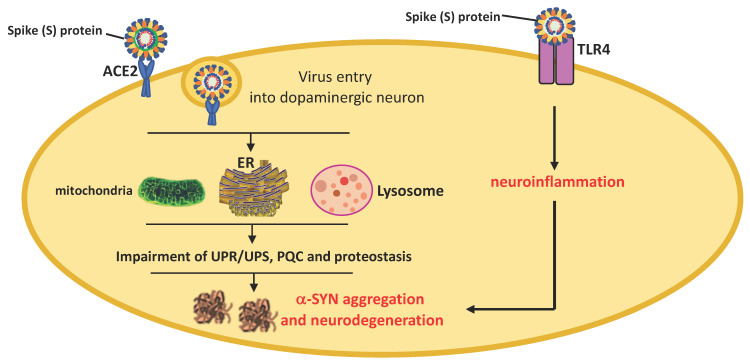
Possible mechanism proposed for α-synuclein aggregation in dopaminergic neurons. The binding of the S protein to the ACE2 receptor mediates the virus entry into dopaminergic cells. In the host cells, the virus can induce endoplasmic reticulum (ER) stress and adaptive unfolded protein response (UPR) and ubiquitin proteasome system (UPS) activation, leading to impairment of proteostasis, α-SYN aggregation, and neurodegeneration. A similar mechanism can be triggered by SARS-CoV-2-induced TLR4 activation.

Furthermore, α-SYN aggregation could also arise from the interaction between SARS-CoV-2 and damaged proteins belonging to autophagy machinery or involved in the maintenance of proteostasis. SARS-CoV-2 was reported to directly bind the human ORF8 protein, causing dysregulation of endoplasmic reticulum trafficking and aberrant proteostasis. This hazardous interaction could be crucial for pathological α-SYN aggregation/accumulation and could be a hindrance for its clearance [172,173,174,175,176] (Figure 5).

Finally, it was recently demonstrated that the expression of ACE2 as well as of other molecules that act as receptors for SARS-CoV-2, including CD209L and sialic acid residues, may be enhanced by interferons and other proinflammatory mediators released following viral infection [34]. Moreover, the interaction between viral proteins and extracellular host proteins can induce conformational changes and accelerate protein aggregation.

## 9. Conclusions

In conclusion, in this review, the possible impact of SARS-CoV-2 infection on the pathogenesis of PD has been discussed. Specifically, the involvement of TLR4 in mediating innate neuroinflammation in the COVID-19 pandemic and subsequent neurodegeneration has been hypothesized. The neuroinvasive potential of SARS-CoV-2 together with the capability to strongly interact with TLR4 through the SARS-CoV-2 S protein suggests an intriguing role for this receptor. A prolonged and dysregulated activation of TLR4 signaling, aside from the possible direct interaction between the SARS-CoV-2 N protein and α-SYN, may impair the protein quality control machinery and host cell proteostasis. The acceleration of the α-SYN aggregation into pathological multimeric protein species such as oligomers/protofibrils increases the vulnerability of nigrostriatal dopaminergic neurons, especially in older people, and leads to widespread neurodegeneration.

Certainly, the long-term link between SARS-CoV-2 infection and PD will be demonstrated when a number of postmortem studies are performed. Moreover, future follow-up of large cohorts of patients with COVID-19 and COVID-19 survivors will be useful to clarify the impact on PD onset and other neurodegenerative disorders.

Although speculative, the role of TLR4 signaling in the COVID-19 pandemic as the fil rouge for the detrimental interconnection between neuroinflammation, α-synuclein accumulation, and dopaminergic cell death deserves to be investigated.

## Data Availability

Not Applicable.
